# Effect of the alveolar type ii cells transplantation for the treatment of acute lung injury

**DOI:** 10.1186/2197-425X-3-S1-A803

**Published:** 2015-10-01

**Authors:** R Guillamat-Prats, F Puig, R Herrero, A Serrano-Mollar, M Camprubí-Rimblas, L Chimenti, J Tijero, MN Gomez, L Blanch, A Artigas

**Affiliations:** Fundació Parc Taulí, Sabadell, Spain; CIBERES (CIBER Enfermedades Respiratorias), Sabadell, Spain; CIBERES (CIBER Enfermedades Respiratorias), Madrid, Spain; IIBB-CSIC, Barcelona, Spain; Corporación Sanitaria y Universitaria Parc Taulí, Critical Care Center, Sabadell, Spain

## Introduction

Acute lung injury (ALI) and Acute Respiratory Distress Syndrome (ARDS) are a clinical manifestation of respiratory failure caused by a response of the lung to local or systemic injury [1]. Damage of alveolar barrier is a critical event in the early stage of ALI/ARDS. Currently there is no effective treatment for this disease. Alveolar type II cells (ATII) are implicated in the alveoli reparation [2] and the transplant of these cells could be a promising ALI treatment.

## Objective

To determine the therapeutic role of ATII cells transplant in an animal model of ALI.

## Methods

Sprague-Dawley (200-250 g) rats were anesthetized with isofluorane. ALI was induced by intratracheal instillation of HCl (0.1mol/L) followed by instillation of LPS from Escherichia coli O55:B5 (30µg/g body weight) 2h later.

(ALI group). Control rats (C) were treated with saline. 9h after the second instillation (LPS instillation), one group of rats were intratracheally transplanted with fresh ATII cells (2.5x10^6^cells) (ATII group). The rats were sacrificed 72h after ALI induction. The effect of ATII cells was assessed by the analysis of bronchoalveolar lavage (cells and protein), proinflammatory markers in lung tissue and histological analysis. Data are expressed as mean ± SEM. 8 animals per group are analyzed. Statistical analysis was performed using One-Way-ANOVA and Newman Keuls post-hoc test. Statistical significance p≤0.05 is considered.

## Results

**Figure 1:**
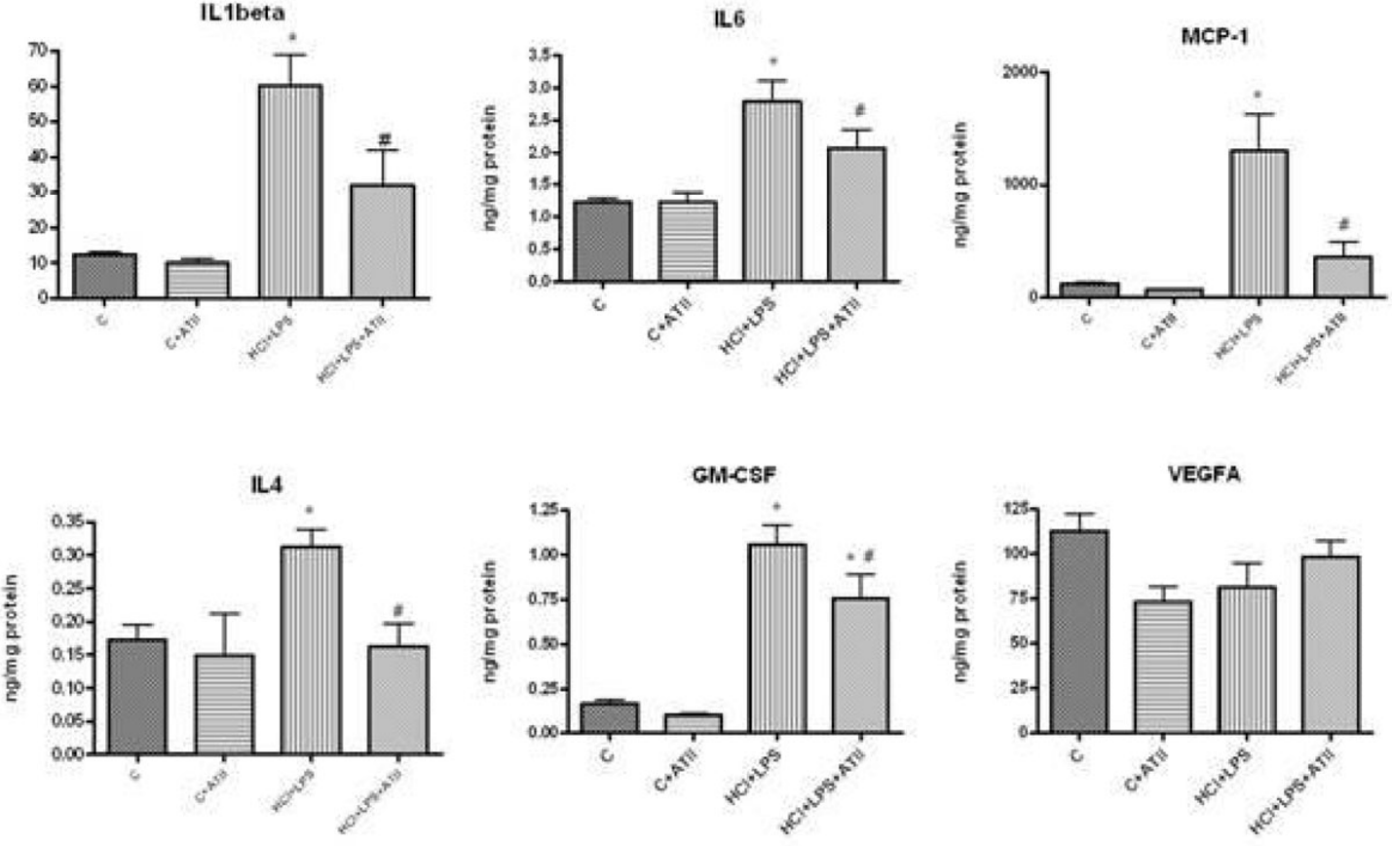
Figure 1

HCl+LPS instillation caused an increase in lung weight owing to the inflammatory component. ATII transplanted group had a significantly reduction in lung weight (C:1,2g ± 0,1; ALI:1.9g ± 0.15, ATII:1.4g ± 0.2). Total protein IgM (C:0.17ng ± 0,01; ALI:0.3ng ± 0.02, ATII:0.19ng ± 0.015) and neutrophil number (C:1% ± 0.5; ALI:54% ± 5, ATII:21% ± 4) in BAL were significantly increased in HCl+LPS group and the ATII cells were able to reduce all of them (p < 0.05) to the control levels. Histological lung tissue analysis showed a recovery of the alveolar damage in ATII transplanted animals versus the peribronchiolar and interstitial infiltration, inflammatory cells, interstitial oedema and haemorrhage observed in ALI rats. The histological score showed significantly differences (C:2 ± 0.5; ALI:8 ± 1, ATII:4 ± 1). Additionaly, proinflammatory (IL1beta and IL6), anti-inflammatory (L4) and cell recruitment markers (MCP-1 and GM-CSF) were evaluated by ELISA in lung homogenates and all of them showed a significantly decrease in ATII cell transplantation group compared to the ALI group (Figure 1).

## Conclusions

The transplantation of ATII cells is able to reduce the pulmonary inflammation and lung injury.

## Grant Acknowledgment

PI12/02548, Fundació Parc Taulí and CIBERES

## References

[1] Ware LB, Matthay MA (2000). The acute respiratory distress syndrome. N Engl J Med.

[2] Guillamat-Prats R, Gay-Jordi G (2014). Alveolar type II cell transplantation restores pulmonary surfactant protein levels in lung fibrosis. J Heart Lung Transplant.

